# Supplementary material for: Time‑resolved multi-omic analysis of paclitaxel exposure in human iPSC‑derived sensory neurons unveils mechanisms of chemotherapy‑induced peripheral neuropathy
Source: Cell Death Dis. 2026 Feb 10;17(1):211. doi: 10.1038/s41419-026-08445-2 (PMC12921266; doi:10.1038/s41419-026-08445-2)
Supplement: Supplementary file 15 — Dataset 1. [file 41419_2026_8445_MOESM15_ESM.zip › CDD_Open_Data/04_RNA_Seq/Timeline_panel_plots_etc_2023-01-23_1609.zip!/Panel_Plots_etc/Evidence_Plots/post5d/evidence_plot_standard contrasts_msigdb_reactome_5d_PTX_vs_DMSO_ID6_M27736_REACTOME_SYNAPTIC_ADHESION_LIKE_MOLECULES.pdf]

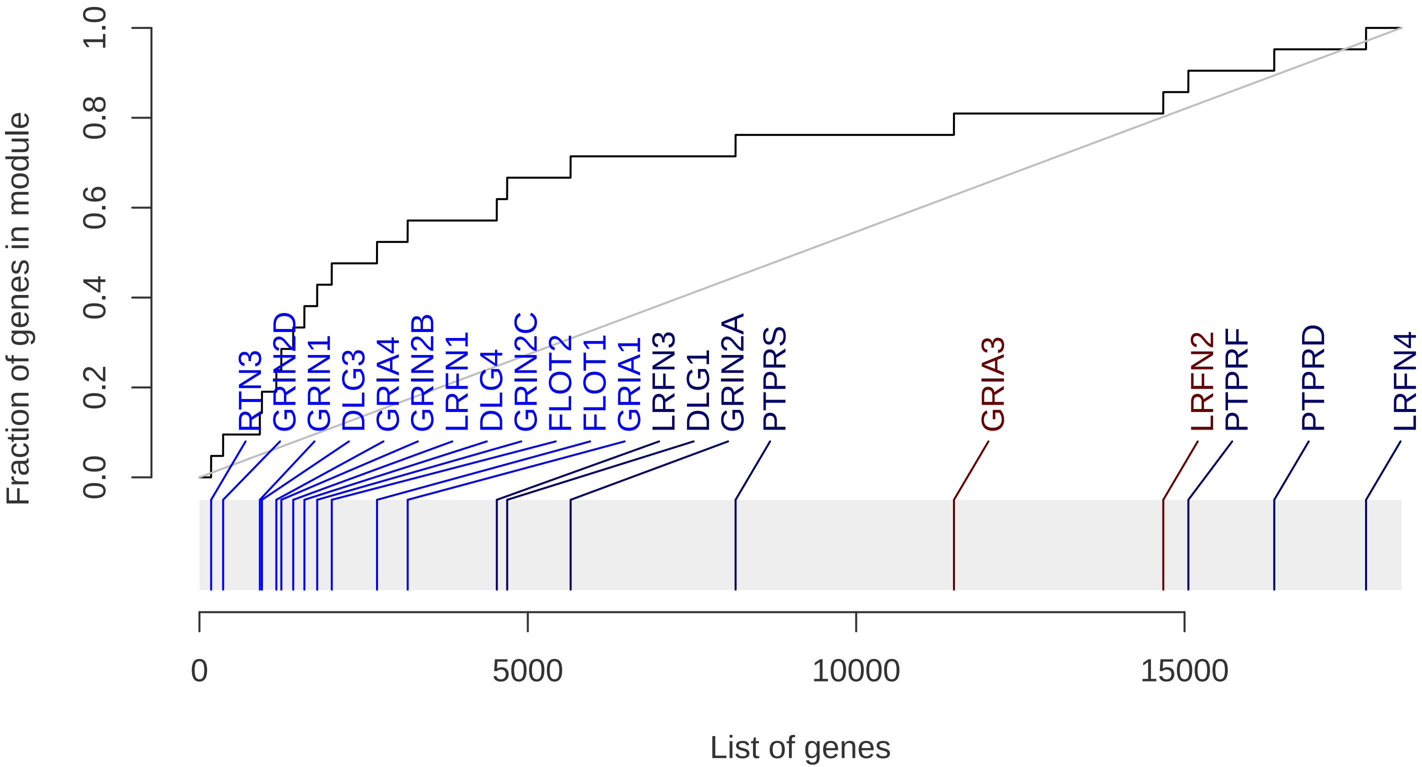

Fraction of genes in module
0.0
0.2
0.4
0.6
0.8
1.0
RTN3
GRIN2D
GRIN1
DLG3
GRIA4
GRIN2B
LRFN1
DLG4
GRIN2C
FLOT2
FLOT1
GRIA1
LRFN3
DLG1
GRIN2A
PTPRS
GRIA3
LRFN2
PTPRF
PTPRD
LRFN4
0
5000
10000
15000
List of genes
